# Prevalence and risk factors of mental health symptoms of individuals in different detention settings: a cross-sectional study

**DOI:** 10.1186/s12888-025-07255-8

**Published:** 2025-09-02

**Authors:** Nina Schnyder, Jérôme Endrass, Joëlle Ninon Albrecht, Jana Dreyer, Marc Graf, Elmar Habermeyer, Astrid Rossegger

**Affiliations:** 1 Department of Psychology, Forensic Psychology Research Group, University of Konstanz, Konstanz, Germany; 2 Research and Development, Office of Corrections and Rehabilitation, Department of Justice and home Affairs, Zurich, Switzerland; 3 Department of Forensic Psychiatry, University of Basel, Basel, Switzerland; 4 Child Development Center, University Children’s Hospital Zurich, University of Zurich, Zurich, Switzerland; 5 Department of Forensic Psychiatry, University Hospital of Psychiatry Zurich, Zurich, Switzerland

**Keywords:** Forensic psychiatry, Epidemiology, Incarcerated individuals, Prevalence, Mental health symptoms, Risk factors, Treatment allocation

## Abstract

**Background:**

The prevalence of mental health symptoms is substantially higher in incarcerated individuals than in the general public. However, little is known how different types of incarceration, including pre-trial and correctional detention as well as detention exclusively for deportation proceedings (administrative detention), are associated with mental health symptoms. We aimed to investigate the prevalence of mental health symptoms in this vulnerable population and examine the impact of different types of detention as well as risk factors on their mental health symptoms.

**Methods:**

Combining two cross-sectional surveys, we assembled a diverse sample of adult individuals incarcerated in the Canton of Zurich, Switzerland. The surveys were conducted in police detention facilities for provisional arrest and in correctional, pre-trial, and administrative detention facilities between July and October 2022 and 2023, respectively. We employed the brief symptom checklist (BSCL) to assess current mental health symptoms, and demographic questionnaires captured relevant risk factors. Logistic regression models were used to examine associations with mental health symptoms.

**Results:**

Of the eligible *N* = 1,868 individuals, *N* = 951 provided sufficient data (completion rate = 50.9%). Our samples were largely representative regarding age, sex, and type of incarceration. Prevalence estimates revealed that almost half of the incarcerated individuals experienced clinically relevant mental health symptoms (44.9%, 95% uncertainty interval (UI): 41.7–48.1%). Risk factors included age, gender, and prior mental health treatment, with younger individuals, female and individuals identifying as non-binary, as well as those who have been treated for a mental health problem before at a two- to threefold greater risk than their counterparts. Types of incarceration demonstrated distinct associations, with individuals in provisional arrest and administrative detention facing a fourfold greater risk.

**Conclusions:**

Our findings underscore the substantial prevalence of mental health symptoms among incarcerated individuals and highlight specific risk factors associated with this vulnerable population. Addressing the mental health needs of female and individuals indentifying as non-binary, and those in provisional arrest or administrative detention is crucial for effective correctional practices. These insights underline the importance of implementing standard screening procedures and tailored interventions to improve mental health outcomes in diverse detention settings.

**Supplementary Information:**

The online version contains supplementary material available at 10.1186/s12888-025-07255-8.

## Background

The prevalence of mental health symptoms and mental disorders is substantially higher in incarcerated individuals than in the general population [1–13]. Several international systematic reviews have been conducted over the past decades. They have shown that the 12-months prevalence of severe mental disorders such as psychotic disorders is around 4% and that of major depression is between 10 and 14% [4, 11]. Furthermore, up to every second newly incarcerated individual has a substance use disorder, and one in four have an attention deficit hyperactivity disorder [9, 10, 13]. Comparably high is the point prevalence of posttraumatic stress disorder which reached up to 21%, with females being at particular risk [12]. These systematic reviews do not include general population samples and compare their estimates to general population surveys and find much larger prevalence estimates. However, different research methodology could make such a comparison challenging. One study used the same method to assess the prevalence of lifetime mood disorders and lifetime substance use disorder both in a prison population as well as in the general population. The prevalence appears to be ten times higher for mood disorders and seven times higher for substance use disorder, within prison populations compared to the general population [6]. The poor mental health of incarcerated individuals is exacerbated by the fact that the comorbidity is very high, with around one in two individuals with a current psychosis or major depression also having a substance use disorder [14]. This heightened burden of disease is exacerbated by emerging evidence indicating that chronic conditions, including mental disorders, receive inadequate treatment within correctional settings [15].

Evidence suggests that mental health states change depending on the prison environment and on the time point of incarceration [16]. Entering prison can have a detrimental effect on mental health, but it improves over time [3, 16]. One population particularly at risk might therefore be pre-trial detainees, as they have typically only recently entered prison [17–20]. Mental disorders are indeed more prevalent in individuals in pre-trial detention, and they are more likely to access mental health care than individuals in other detention settings [17–19]. Furthermore, remand individuals might have more difficulties adjusting to the prison environment as subjective well-being decreases [20]. It has been argued that situational stressors related to the uncertainty of their trial might be associated with their difficulties to adapt to prison [21]. Another population that might be particularly at risk for poor mental health could be those detained exclusively for deportation proceedings as the inherent uncertainty of their situation might be an important stressor. However, evidence on individuals awaiting deportation is scarce. Emerging evidence from the USA suggests that the threat of or worries about a potential deportation are associated with psychological distress and worse mental health [22, 23].

Given the high prevalence of mental health problems in prison populations, providing access to primary mental health care is necessary. Currently in Switzerland, individuals are given access to primary mental health care if they express a need for it or if a correctional officer files a request. The former assumes that individuals are self-aware of mental health needs and know how to access care. The latter assumes that correctional officers can identify symptoms of mental disorders. However, mental disorders can manifest themselves in different ways which presents a substantial challenge in detection, potentially leading to the oversight of affected individuals. Consistent with World Health Organization recommendations, systematic screening is essential for identifying those at risk [24–26], but it is usually not done in Switzerland.

The present study pursued three objectives. First, it estimated the prevalence of common mental-health symptoms among incarcerated individuals in Switzerland. Second, it compared the distribution of these symptoms across distinct detention contexts - remand, sentenced imprisonment, and detention pending deportation - thereby extending earlier work that has linked detention type to mental-health outcomes [1, 16, 17]. To our knowledge, no prior study has reported prevalence estimates simultaneously for all three settings. Third, it examined key correlates of mental-health problems, including gender and previous mental healthcare utilisation, with the aim of identifying priority groups for targeted interventions.

To address these aims, we conducted a cross-sectional, observational epidemiological survey of a diverse sample of individuals held in the aforementioned detention settings. Specifically, our research questions were:

1. What is the prevalence of mental-health symptoms among incarcerated individuals in Switzerland?
2. Which common risk factors (including sociodemographic and incarceration-related factors) are associated with these symptoms?
3. Does the occurrence of mental-health symptoms differ by type of incarceration (remand, sentenced, or awaiting deportation)?

## Methods

### Settings

Our study combines two cross-sectional surveys into a unique sample of adult individuals of diverse genders who were incarcerated in the Canton of Zurich, Switzerland.

In survey 1, we recruited individuals in provisional arrest at a police detention facility from 2022-07-26 to 2022-10-31. All individuals have been very recently arrested, usually a few hours ago. Detainees were in their cells for up to 23 h each day. We visited the facility each morning from Monday to Friday during the three-month study period (see online material 1 for more details).

In Survey 2, we recruited remand or sentenced individuals at nine correctional and pre-trial detention facilities, as well as individuals in administrative detention, from 2023-07-03 to 2023-10-19. Individuals in correctional facilities have been convicted, and those in pre-trial detention await sentencing or trial. There are separate correctional facilities for shorter (up to 1.5 years) and longer (1.5 years or longer) sentences. Individuals in administrative detention are not convicted of a criminal offence but await deportation from Switzerland. Compared to correctional and administrative detention, individuals in pre-trial detention spend the greatest amount of time confined to their cells. We visited each facility at least twice (see online material 1 for more details).

### Procedures

During the study period of Survey 1, we received a daily list of all individuals held on remand at the participating facility. We approached all eligible individuals who had been arrested within one to two days prior, over the course of the three-month data collection period. For Survey 2, we obtained a list of all incarcerated individuals present at each facility on the first day of our visit and approached all eligible participants from these lists. From both surveys, we extracted basic demographic and incarceration-related information (sex, age, and type of incarceration) for the total eligible population. This enabled us to assess the representativeness of our final sample with respect to these characteristics.

Participants completed a self-report paper-pencil questionnaire on their current mental health symptoms. To maximise response rate and minimise response bias, key strategies have been employed in both surveys. The study documents were provided in languages that are commonly spoken and understood in Zurich’s prison and jail population (German, English, French, Italian, Spanish, Portuguese, Arabic, Croatian, Albanian, Serbian, Macedonian, Romanian, and Turkish). The limited proficiency in, or inadequate comprehension of the languages that could be effectively utilised by us during interviews made the use of paper-pencil questionnaires necessary (see online material 1 for more details). If an individual could not read or write and we had sufficient time, we read the questionnaire to them. Individuals were allowed to help each other to complete the questionnaire. To minimise potential self-selection bias and increase response rates, we implemented two strategies: First, we provided small incentives to encourage participation. In Survey 1, participants received chocolate or candy, while in Survey 2, they were given CHF 10.– vouchers redeemable at the facility kiosk. Monetary vouchers were not offered in Survey 1 due to the absence of a kiosk at that site. Second, the study team established direct personal contact with all eligible individuals, actively encouraging participation and addressing any questions or concerns raised during the recruitment process.

Data was entered into REDCap [27] by one person, and entries were reviewed and, if necessary revised, by another. The reporting of this study follows the “STrengthening the Reporting of OBservational studies in Epidemiology” (STROBE) statement [28].

### Participants

In both surveys, all adults (min. 18 years old) who could give written informed consent and understand one of the provided languages were eligible to participate. Exclusion criteria for the surveys included incapacity to provide informed consent, compromised decision-making ability, risk of unsafe interaction with study procedures, documented severe illness (such as acute psychosis or suicidality), or scheduled release from the facility on the day of our visit. Additionally, individuals had to be present during our visits.

### Measures

#### Psychological distress

We used the brief symptom checklist (BSCL), the latest version of the brief symptom inventory (BSI), to measure current mental health problems and symptoms of mental disorders (further referred to as mental health symptoms) in the past seven days [29, 30]. The BSCL is a validated self-report measure, exists in different languages, and has been used in a variety of populations, including remand and sentenced individuals [3, 31–33]. The BSCL is commonly used as screening in clinical settings but can also be used in epidemiological studies [30]. Participants are asked how much they have been distressed by 53 physical and mental health-related symptoms in the past seven days. Answers are recorded on a 5-point Likert scale ranging from 0 = “not at all” to 4 = “extremely”.

Symptoms are categorised into nine dimensions: anxiety, depression, hostility, interpersonal sensitivity, obsession-compulsion, paranoid ideation, phobic anxiety, psychoticism, and somatic complaints. Four symptoms are not used for the categorisation but are of clinical importance (e.g. problems sleeping). Furthermore, the global severity index (GSI), based on all 53 symptoms, indicates the overall level of mental health symptoms.

Since we needed the BSCL in languages other than German, we contacted authors who had translated and used the BSCL before. The BSCL or some of its items were unavailable in Arabic, Romanian, Spanish, and Turkish. Professional translators translated the German version into the four target languages. Other translators, blind to the original version, translated it back to German. We reviewed the back-translated German version and asked the service to re-translate, if necessary.

#### Type of incarceration

We assessed types of incarceration accounting for (a) how recently someone has been detained, (b) the typical length of the detention period, (c) the verdict received or awaiting trial, and (d) criminal offence or non-criminal violation. This led to five types of detention based on criminal violation: (1) provisional arrest (very recently detained individuals without a verdict); (2) pre-trial detention (detained individuals awaiting trial); (3) shorter incarceration (individuals with prison sentences up to 1.5 years); (4) longer incarceration (individuals with prison sentences of 1.5 years or longer); and an additional one for a non-criminal violation: (5) administrative detention (individuals exclusively detained for deportation proceedings).

#### Demographic characteristics and potential risk factors

We assessed age (groups < 20, 20–29, 30–39, 40–49, 50–59, > 60 years), gender (male, female, non-binary), nationality (Swiss or non-Swiss), currently being in a relationship (yes or no), ever having been treated for a mental disorder (yes or no), and ever having been remand or sentenced before (yes or no). These risk factors were selected purposefully, as they might have clinical importance for mental health symptoms. Demographic characteristics and the remaining study documents were professionally translated by one and reviewed by two independent translators.

### Statistical analyses

#### Representativeness of samples

Since Survey 1 was conducted anonymously, we did not identify which specific eligible individuals responded. We evaluated representativeness by comparing the age and gender distribution of eligible individuals with those of the respondents (see files RepresentativeSamples_EvalPG_GZW_240111 on OSF). Conversely, Survey 2 allowed for the assessment of individual responses. We assessed representativeness using three univariable logistic regressions, with response (yes/no) as the dependent variable and age, sex, and type of detention as independent variables. In both surveys, we were not able to analyse representativeness of gender including the category non-binary as this is not recorded.

#### Missing data

As recommended by the BSCL manual, we have analysed whether data was missing at random to understand patterns of missing data [30]. First, we summarised each item and examined how many missings were on each item. Second, we examined missing value maps to investigate patterns [34]. Third, we examined missing patterns for each of the nine symptom dimensions and the additional items to further investigate potential patterns [34] (see files 02_MissingDataBSCL_231114 on OSF). The manual allows a maximum of one missing item per dimension and a maximum of 13 missing items for the global severity index (GSI). Individuals with more missing data were excluded from analyses.

#### BSCL norms and case definition

We followed the German manual to analyse and interpret the BSCL [30]. First, we calculated mean sum scores for each of the nine dimensions and for the GSI (total scale). Applying prorated scale scoring, available items were averaged [30, 35]. Second, we transformed mean sum scores into standardised *T*-values (mean = 50, standard deviation (SD) = 10) using the provided norms of the representative German population sample [30] (Appendix B of the BSCL manual, pp. 106). We used gender-specific norms. For a lack of norms for those who had identified as non-binary, we used once the female and once the male norms. Our classification did not differ no matter which norms were used. The BSCL manual defined clinically significant mental health symptoms that need further attention of a mental health professional in two ways: [1] *T*-value of GSI > = 63 or [2] *T*-values of min. two dimensions > = 63. This is standard when identifying a “case” with the BSCL. We followed this standard and categorised each individual as a case, both based on the GSI and on two dimensions (0 = no, 1 = yes).

#### Calculation of prevalence and 95% uncertainty interval (UI)

Two measures of the prevalence of mental health symptoms in the past seven days were calculated based on [1] the GSI (total sum score) and [2] two symptom dimensions. For each dimension, we also calculated how many individuals have values above the pre-defined cut-off score of T > = 63. Prevalence and corresponding 95% UI were estimated with the Wilson score interval recommended for larger and smaller samples [36–38].

#### Modelling

For modelling, the case of mental health symptoms was based on the BSCL total sum score. We modelled associations between risk factors and mental health symptoms, as well as between type of incarceration and mental health symptoms with logistic regressions. To avoid overfitting, risk factors were included in the models only if their *p*-value, as determined by the chi-square test in the univariable analyses, was less than 0.25 [39]. We examined interaction terms for risk factors based on statistical and clinical considerations. They were only added in the adjusted models if statistically significant in the univariable analyses [39]. The independent variable and covariates were entered simultaneously into the models. We evaluated the models’ goodness-of-fit with McFadden pseudo-*R*^2^ and le Cessie-van Houwelingen normal test statistic for the unweighted sum of squared errors [39–42]. Furthermore, we used Wald test to evaluate the statistical significance of each variable [39].

All analyses were done in R (version 4.3.2) and RStudio (version 2023.06.1 + 524), using the packages tidyverse, janitor, kableExtra, epiR, DescTools, survey, and rms [38, 43–48]. The publication was written and compiled in R Markdown [49].

### Ethics statement

The ethics commission of the Canton of Zurich has approved the first survey on July 12 2022 (BASEC-Nr.: 2022 − 00887), and the second on June 19 2023 (BASEC-Nr.: 2023 − 00697).

## Results

### Completion rate, missing data, and representativeness of samples

**Fig. 1:**
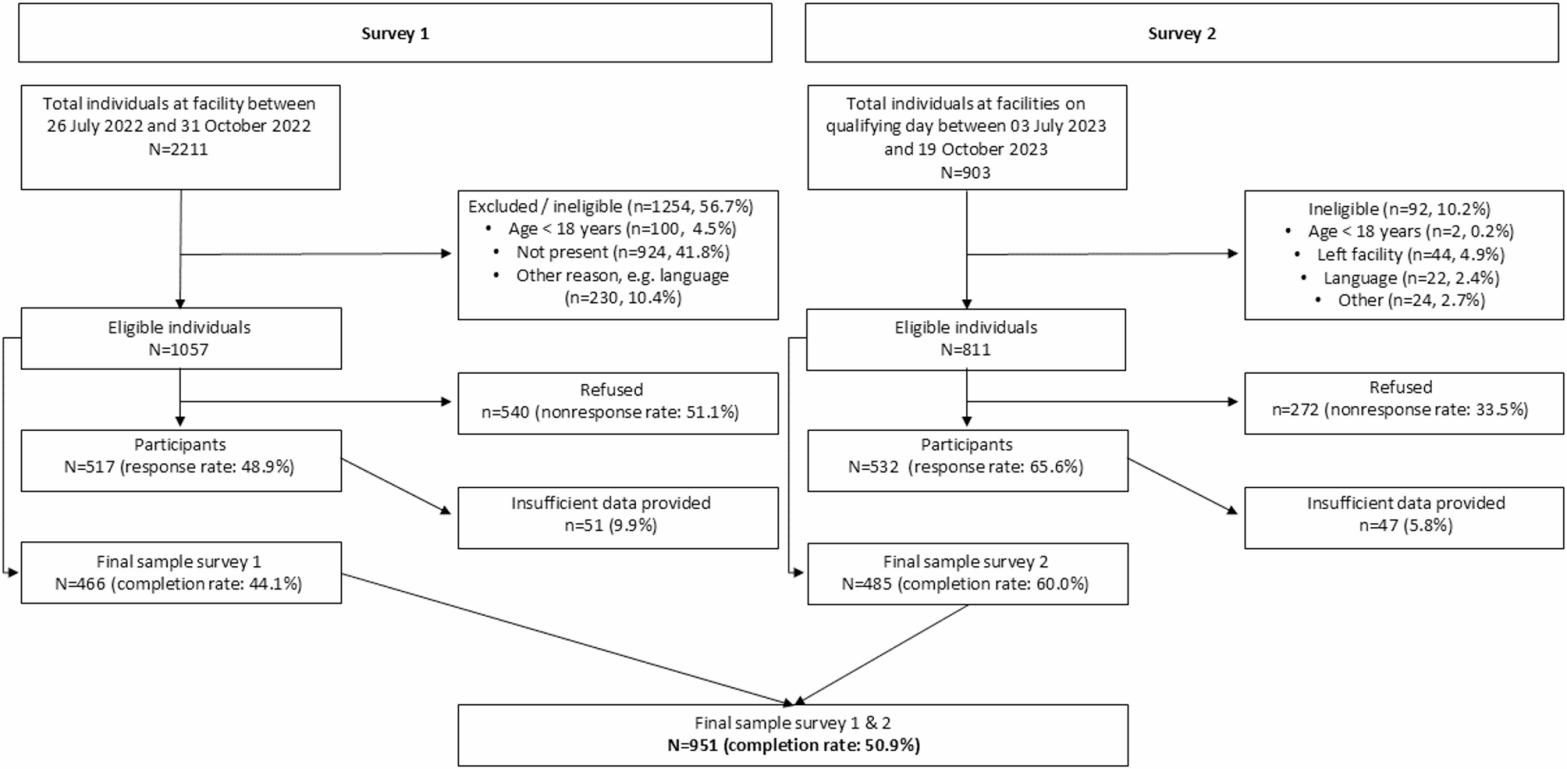
STROBE flow-chart: response and completion rate of the two surveys

A total number of *N* = 1,868 were eligible to participate in the two surveys. Of these, *N* = 1,049 gave consent (response rate = 56.2%), and *N* = 951 provided sufficient data (completion rate = 50.9%) (Fig. 1).

For each dimension, between 924 (97.2%) (depression) and 943 (99.2%) (anxiety) provided sufficient data (eTable 1). Data was missing at random (see files 02_MissingDataBSCL_231114 on OSF). The interpretation of the BSCL is not impeded.

Both survey samples 1 and 2 were representative in terms of age and sex for all eligible individuals. However, there was a slight response bias in survey 2, with individuals in pre-trial detention being more likely to respond than individuals with shorter or longer prison sentences, or those in administrative detention.

### Sample characteristics

Our sample consisted predominantly of males between the ages 20 and 49. Slightly more than half had been on remand or sentenced before, and slightly less than half were in a relationship at the time of the surveys. One in three individuals had received treatment for a mental health problem previously, and three out of ten were Swiss. The largest proportion of the sample was in provisional arrest, followed by longer incarceration and pre-trial detention. The smallest proportion was in administrative detention (Table 1; eTable 2 for the questionnaire’s language distribution).

**Table 1:** Sample characteristics and prevalence of clinically relevant mental health symptoms (*N* = 951)

		Sample characteristics	Prevalence of mental health symptoms based on GSI			Prevalence of mental health symptoms based on two dimensions		
		% (*n*)	%	Lower UI	Upper UI	%	Lower UI	Upper UI
Total sample	total sample	100% (951)	44.9	41.7	48.1	55.0	51.8	58.1
Type of incarceration	provisional arrest	49.0% (466)	57.6	53.0	62.0	66.7	62.3	70.9
	pre-trial detention	17.9% (170)	33.5	26.8	41.1	45.9	38.6	53.4
	shorter incarceration	8.5% (81)	36.7	26.9	47.7	45.7	35.3	56.5
	longer incarceration	21.8% (207)	27.2	21.6	33.7	39.6	33.2	46.4
	administrative	2.8% (27)	57.7	38.9	74.5	55.6	37.3	72.4
Age group	<20	4.5% (42)	59.5	44.5	73.0	71.4	56.4	82.8
	20–29	33.4% (312)	48.2	42.7	53.8	59.6	54.1	64.9
	30–39	32.0% (299)	46.3	40.6	52.0	56.2	50.5	61.7
	40–49	17.8% (166)	36.8	29.8	44.4	47.6	40.1	55.2
	50–59	8.3% (78)	31.2	21.9	42.2	39.7	29.6	50.8
	>=60	4.1% (38)	56.8	40.9	71.3	60.5	44.7	74.4
Gender	female	7.0% (65)	64.6	52.5	75.1	73.8	62.0	83.0
	male	91.4% (846)	42.8	39.5	46.1	53.2	49.8	56.5
	non-binary	1.6% (15)	80.0	54.8	93.0	80.0	54.8	93.0
Nationality Swiss	no	70.0% (651)	45.4	41.6	49.3	55.5	51.6	59.2
	yes	30.0% (279)	43.5	37.8	49.4	54.1	48.3	59.9
Currently in a relationship	no	54.6% (502)	43.8	39.5	48.2	54.0	49.6	58.3
	yes	45.4% (417)	46.5	41.7	51.3	56.8	52.0	61.5
Previous treatment for mental health problems	no	65.2% (606)	35.1	31.4	39.0	45.0	41.1	49.0
	yes	34.8% (323)	63.7	58.2	68.8	74.3	69.3	78.8
Previous incarceration	no	41.3% (381)	45.6	40.6	50.6	54.9	49.8	59.8
	yes	58.7% (541)	44.4	40.3	48.6	55.3	51.1	59.4

### Prevalence of clinically relevant mental health symptoms

#### Total sample

Prevalence estimates were higher when based on two symptom dimensions than when based on the GSI (total sum score of the BSCL). According to the GSI, slightly less than one in two individuals had experienced clinically relevant mental health symptoms in the past seven days, while based on the two symptom dimensions, slightly more than one in two had (Table 1).

#### Stratified sample

Examining and comparing prevalence across different sub-samples showed that the largest proportion of individuals with mental health symptoms were in provisional arrest and administrative detention, followed by those in shorter incarceration, pre-trial detention, and longer incarceration. Additionally, younger individuals (aged < 20–39) and older individuals (aged > = 60) appear to experience mental health symptoms more frequently than middle-aged individuals (aged 40–59). Those identifying as female or non-binary have higher rates of mental health symptoms than those identifying as males, with three in five and four in five, respectively, being affected. Similarly, individuals who had previously received mental health care also seem more affected. Regarding characteristics such as nationality, current relationship status, or prior incarceration, there appears to be no significant difference in the prevalence of mental health symptoms (Table 1).

#### Dimensions

**Fig. 2:**
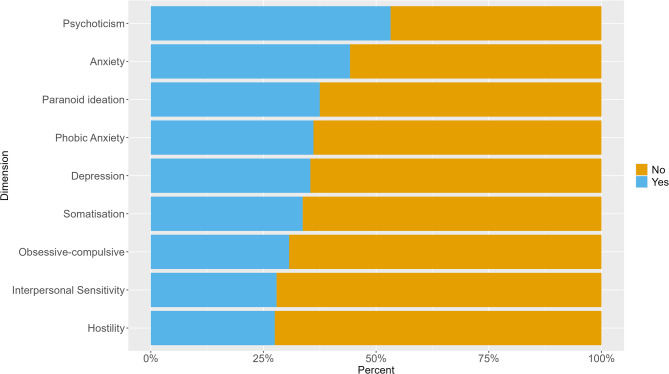
Mental health symptoms above cut-off (total sum score) by dimensions

Mental health symptoms in the dimensions of psychoticism (e.g. “the idea that someone else can control your thoughts” or “feeling lonely even when you are with people”) and anxiety (e.g. “nervousness or shakiness inside” or “feeling fearful”) were reported by the largest proportion of individuals. In contrast, hostility (e.g. “feeling easily annoyed or irritated” or “having urges to beat, injure, or harm someone”) and interpersonal sensitivity (e.g. “your feelings being easily hurt” or “feeling that people are unfriendly or dislike you”) were reported by the smallest proportion (Fig. 2).

### Risk factors for mental health symptoms

Based on univariable analyses, only age, gender, and previous treatment for mental health symptoms were included in the adjusted model. Nationality, relationship status, and previous incarceration were not included. Interactions between risk factors were either not statistically significant or could not be computed due to small sample sizes in some cells (see files 04_PrevalenceBSCL_afterPR_241017 on OSF for more details).

In the adjusted model, individuals aged 40–49 and 50–59 were less likely to experience mental health symptoms compared to those aged 20–29 (the reference category). Additionally, individuals identifying as female or non-binary, and those who had previously been treated for a mental health problem, were more likely to experience symptoms than their counterparts (Table 2). The model fit the data well.

**Table 2:** Association between risk factors and case of mental health symptoms (*N* = 951)

		OR	Lower 95%-UI	Upper 95%-UI
Age in years	<20	1.753	0.887	3.524
	30–39	0.933	0.664	1.311
	40–49	0.613	0.406	0.922
	50–59	0.496	0.281	0.858
	>= 60	1.507	0.734	3.135
Gender	female	2.136	1.226	3.806
	non-binary	3.520	1.030	16.222
Previous treatment for mental health problems	yes	3.202	2.396	4.298

*OR *Odds Ratio, *UI* uncertainty interval. Model adjusted for age, sex, and previous treatment for mental health problems. Reference categories: age 20-29, male gender, not previously treated for mental health problems. McFadden Pseudo *R*^2^ (adjusted model)=0.079; le Cessie-van Houwelingen test *p*=0.749; Wald test: all covariates significantly contribute to model fit

### Association between type of incarceration and mental health symptoms

Compared to individuals with longer prison sentences, those in administrative detention and in provisional arrest were four times more likely to experience mental health symptoms. Individuals in pre-trial detention and those with shorter or longer prison sentences had comparable odds to experience clinically relevant mental health symptoms (Table 3; for unadjusted estimates and all estimates of the fully adjusted model see eTable3). The model fitted the data well.

**Table 3:** Association between type of incarceration and case of mental health symptoms (*N* = 951)

		OR	Lower 95%-UI	Upper 95%-UI
Type of detention	administrative detention	4.842	1.926	12.504
	pre-trial detention	1.509	0.924	2.474
	provisional arrest	4.294	2.877	6.506
	shorter incarceration	1.584	0.859	2.900

*OR* Odds Ratio, *UI* uncertainty interval. Model adjusted for age, gender, and previous treatment for mental health symptoms. Reference category: longer incarceration. McFadden Pseudo *R*^2^ (adjusted model) = 0.135; le Cessie-van Houwelingen test *p* = 0.950; Wald test: all covariates significantly contribute to model fit

## Discussion

In prison populations, the prevalence of mental disorders is high, and prisoners report high levels of mental health symptoms. Our study was the first to quantify and contrast the mental health challenges across different prison populations including individuals in police custody and immigration detention. It also contributes to the limited evidence on mental health symptoms of remand and sentenced individuals in one jurisdiction in Switzerland. Furthermore, our study gives insights into risk factors for mental health symptoms in prison populations. It has important implications in how to potentially adapt and improve correctional practice.

Our study had three main findings. First, using the screening tool Brief Symptom Checklist to identify clinically relevant levels of mental health symptoms, almost half of all incarcerated individuals were found to experience them. This represents an almost 500% increase over the levels of problems experienced by the general population where one in ten experience comparable levels of symptoms [30]. Prevalence estimates of mental health symptoms in incarcerated populations can vary depending on the screening tool used. Nevertheless, our estimates align with international evidence reporting prevalence rates of 40–60% for depressive symptoms, posttraumatic stress disorder symptoms, or any current severe symptoms among prison populations [50–54]. A study from the Netherlands that used the same instrument as our study reported lower rates, though they did not apply the same normative criteria as they have used Dutch norms [3]. The prevalence might be slightly underestimated in our study, as we had to exclude individuals with compromised decision-making ability. The severity of reported symptoms, including psychoticism, anxiety, and paranoid ideation, warrants intervention by mental health professionals, underscoring the heightened vulnerability of this cohort [26].

Second, adults up to 40, females, and those identifying as non-binary, as well as individuals with a history of treatment for a mental health problem were at two- to threefold greater risk of experiencing mental health symptoms than their counterparts. A high prevalence of poor mental health in young offenders, especially juveniles, has been found before [55–58]. At the same time, it has been previously recommended that special attention should be paid to individuals who enter detention at age 30 or above because these individuals seem to have an elevated need for mental health care [3], which is only partly supported by our evidence. Our evidence suggests that individuals up to 40 should be particularly focused on. Supporting our findings, previous research has consistently recognised females as a particularly vulnerable group within incarcerated populations, which is comparable to findings from the general population [59–63]. Preliminary findings suggest that severe mental health symptoms such as delusions at the time of the offence might be more likely in female than male offenders [62]. In line with our findings, emerging evidence suggests that transgender inmates have a higher risk of post-traumatic stress disorder, depression, and suicide attempts than their counterparts [64]. In Switzerland, individuals who identify as non-binary are housed in the general prison population. At the time of our study, only one incarcerated individual was registered as transgender. This individual was housed in the population they identified with. While there is a risk of neglecting gender differences in the context of incarcerated individuals due to the predominant male demographic, our findings indicate that it is crucial to shift attention towards addressing the mental health care needs of female, and potentially non-binary, offenders. Our findings on age and gender both highlight the importance of directing policy focus towards these specific demographics. Additional evidence is required for individuals identifying as non-binary, given the limited sample size in our study. Information on risk factors could be useful in service planning at both detention and correctional centres, guiding effective resource allocation strategies.

Third, at particular high risk of experiencing mental health symptoms are remand individuals very shortly after they have been arrested (provisional arrest), and individuals awaiting deportation. There are several possible explanations for this. Different types of mental disorders are discussed to be a risk factor for violent criminal behaviour [65–67] and symptoms might therefore be highest right after the arrest. Regarding remand individuals, mental health symptoms might improve over the course of detention [3]. This improvement might be associated with the detention regime itself. In Zurich, detention regime right after arrest is strict, with individuals being able to spend only one hour outside their cells and having only very limited contact with peers, family, and friends. Later on in pre-trial detention, the regime is less strict; up to eight hours can be spent outside the cells, and interpersonal contact is much less limited. Furthermore, the initial phase of detention might be very stressful as individuals are cut off from their relatives and other important contacts, have to cope with insecurity regarding their future and potentially unsafe environments. The level of mental health symptoms might decline after the adjustment period. It is currently unclear whether mental health symptoms remitted spontaneously or whether they improved because mental health care was provided. Evidence on individuals awaiting deportation is very limited. This type of detention might have adverse effects on mental health. Levels of mental health symptoms in this population are very high, time in detention is positively associated with problems, and the negative impact of detention on mental health persists even after release [68, 69]. The planning and provision of mental health care for this group may pose particular challenges related to cultural aspects and language skills. Nevertheless, our study underscores the importance of policymakers redirecting their focus to address the needs of this specific group of detainees.

Our study provides important findings of mental health symptoms in incarcerated individuals. A limitation of the cross-sectional survey methodology of our study is that we do not know why individuals experience mental health symptoms. Particularly among recently arrested individuals, we do not know whether individuals have already had mental health symptoms before arrest, or if their problems are a reaction to the detention. This limitation is negligible for the scope of our study, as the timing of symptom onset does not impact resource planning and allocation. We currently do not know whether individuals who need mental health care receive needed care. This should be investigated in the future. Additionally, while self-reported screenings neither allow for the determination of a diagnosed mental disorder nor the intensity of care required, they do provide an estimation of the need for crisis intervention or ongoing support within the correctional setting. Importantly, we had decided to use a self-report measure for two reasons. First, a large proportion of incarcerated individuals in Switzerland do not speak sufficient German, French, or English to conduct structured or semi-structured diagnostic interviews. Second, to investigate usability of mental health screenings in Swiss prisons. The slight response bias regarding type of detention is negligible as the sample is representative regarding other assessed parameters and the results are stratified by type of detention. Future studies should, however, focus on individuals awaiting deportation as our sample size for this population was small. The present study was not designed to examine ethnical or racial differences regarding mental health symptoms. Future studies should focus on this as ethnical and racial minorities might face distinct barriers to accessing mental health services within correctional settings. Furthermore, future research should consider incorporating additional key risk factors for mental health problems, such as socioeconomic deprivation, experiences of homelessness, and substance use-related issues.

## Conclusions

Incarcerated individuals are in a very vulnerable psychological state. The present study’s findings suggest that, depending on the type of incarceration, up to three out of five incarcerated individuals report such pronounced mental health symptoms that an examination by and support from a mental health specialist is indicated. Prison staff are confronted with a demanding and highly stressed population. The findings of our study underline that the World Health Organization’s recommendation to implement screenings should upon entry to prison should be followed. This could help identify vulnerable individuals early and optimize the allocation of staff resources [70].

The findings have important implications for practice. First, sufficient staff should be at the facility to enable conversations and interactions with particularly vulnerable detainees. Those with clinically relevant levels of mental health symptoms would benefit from care provided by specialised healthcare professionals including, e.g., psychologists or psychiatric nurses. Second, staff should be trained to provide people with the support they need. Third, as the most common dimensions of mental health symptoms were psychoticism, anxiety, and paranoid ideation, it appears that correctional officers may need continuing education opportunities in working with persons experiencing suspicion and panic. Until such measures are put in place, clinically relevant levels of mental health symptoms will represent a barrier to successful rehabilitation and re-integration to society.

## Supplementary Information

Supplementary Material 1.

## Data Availability

The datasets generated and/or analysed during the current study are not publicly available due ethical reasons (information on a very vulnerable population) but are available from the corresponding author on reasonable request. Researchers can apply for the data with the corresponding author by submitting a study protocol. R scripts and the data dictionary codebooks are available online on the Open Science Framework (OSF): https://osf.io/cy6b7/.

## Abbreviations

BASEC: Business Administration System for Ethics Committees
BSCL: Brief Symptom Checklist
BSI: Brief Symptom Inventory
GSI: Global Severity Index
OR: Odds Ratio
OSF: Open Science Framework
SD: Standard Deviation
STROBE: STrengthening the Reporting of OBservational studies in Epidemiology
UI: Uncertainty Interval

## References

[1] Brinded PMJ, Simpson AIF, Laidlaw TM, Fairley N. Prevalence of psychiatric disorders in New Zealand prisons: a national study. Aust N Z J Psychiatry. 2001;35(2):166–73. 11284897 10.1046/j.1440-1614.2001.00885.x

[2] Butler T, Allnutt S, Cain D, Owens D, Muller C. Mental disorder in the New South Wales prisoner population. Aust N Z J Psychiatry. 2005;39(5):407–13. 15860030 10.1080/j.1440-1614.2005.01589.x

[3] Dirkzwager AJE, Nieuwbeerta P. Mental health symptoms during imprisonment: a longitudinal study. Acta Psychiatr Scand. 2018;138(4):300–11. 30003548 10.1111/acps.12940

[4] Fazel S, Seewald K. Severe mental illness in 33,588 prisoners worldwide: systematic review and meta-regression analysis. Br J Psychiatry. 2012;200(5):364–73. 22550330 10.1192/bjp.bp.111.096370

[5] Heffernan EB, Finn J, Saunders JB, Byrne G. Substance-use disorders and psychological distress among police arrestees. Med J Aust. 2003;179(8):408–11. 14558863 10.5694/j.1326-5377.2003.tb05617.x

[6] Macciò A, Meloni FR, Sisti D, Luigi Rocchi MB, Petretto DR, Masala C. u. A. Mental disorders in Italian prisoners: results of the redime study. Psychiatry Res. 2015;225(3):522–30. 25534756 10.1016/j.psychres.2014.11.053

[7] Nielssen O, Misrachi S. Prevalence of psychoses on reception to male prisons in New South Wales. Aust N Z J Psychiatry. 2005;39(6):453–9. 15943646 10.1080/j.1440-1614.2005.01603.x

[8] Sirdifield C, Gojkovic D, Brooker C, Ferriter M. A systematic review of research on the epidemiology of mental health disorders in prison populations: A summary of findings. J Forensic Psychiatry Psychol. 2009;20(Sup1):78–101.

[9] Fazel S, Bains P, Doll H. Substance abuse and dependence in prisoners: a systematic review. Addiction. 2006;101(2):181–91. 16445547 10.1111/j.1360-0443.2006.01316.x

[10] Young S, Moss D, Sedgwick O, Fridman M, Hodgkins P. A meta-analysis of the prevalence of attention deficit hyperactivity disorder in incarcerated populations. Psychol Med Januar. 2015;45(2):247–58. 10.1017/S0033291714000762 PMC4301200 25066071

[11] Fazel S, Danesh J. Serious mental disorder in 23 000 prisoners: a systematic review of 62 surveys. Lancet. 2002;359(9306):545–50. 11867106 10.1016/S0140-6736(02)07740-1

[12] Baranyi G, Cassidy M, Fazel S, Priebe S, Mundt AP. Prevalence of posttraumatic stress disorder in prisoners. Epidemiol Rev. 2018;40(1):134–45. 29596582 10.1093/epirev/mxx015 PMC5982805

[13] Fazel S, Yoon IA, Hayes AJ. Substance use disorders in prisoners: an updated systematic review and meta-regression analysis in recently incarcerated men and women. Addiction. 2017;112(10):1725–39. 28543749 10.1111/add.13877 PMC5589068

[14] Baranyi G, Fazel S, Langerfeldt SD, Mundt AP. The prevalence of comorbid serious mental illnesses and substance use disorders in prison populations: a systematic review and meta-analysis. Lancet Public Health. 2022;7(6):e557–68. 35660217 10.1016/S2468-2667(22)00093-7 PMC9178214

[15] Curran J, Saloner B, Winkelman TNA, Alexander GC. Estimated use of prescription medications among individuals incarcerated in jails and state prisons in the US. JAMA Health Forum. 2023;4(4):e230482. 37058293 10.1001/jamahealthforum.2023.0482 PMC10105311

[16] Walker J, Illingworth C, Canning A, Garner E, Woolley J, Taylor P, Amos T. Changes in mental state associated with prison environments: a systematic review. Acta Psychiatr Scand. 2014;129(6):427–36. 10.1111/acps.12221 24237622

[17] Gerth J, Endrass J, Weber M, Graf M, Singh JP, Rossegger A. Exploring the mental healthcare needs of Swiss pre-trial detainees: a pilot investigation of an on-site psychiatric day clinic. Front Psychiatry. 2022;13:924861. 35928770 10.3389/fpsyt.2022.924861 PMC9345322

[18] Spycher J, Dusheiko M, Beaupère P, Gravier B, Moschetti K. Healthcare in a pure gatekeeping system: utilization of primary, mental and emergency care in the prison population over time. Health Justice. 2021;9(1):11. 10.1186/s40352-021-00136-8 PMC8120814 33987749

[19] Andrade J, Sousa M, Gonçalves RA, Castro-Rodigues A. Remand prisoners’ specific needs: A systematic review. J Police Crim Psychol. 2023;38(4):942–55.

[20] Bloem O, Bulten E, Verkes RJ. Changes in subjective wellbeing of prisoners on remand. Int J Prison Health. 2019;15(2):181–91. 31172856 10.1108/IJPH-01-2018-0003

[21] Dahle KP, Lohner JC, Konrad N. Suicide prevention in penal institutions: validation and optimization of a screening tool for early identification of high-risk inmates in pretrial detention. Int J Forensic Ment Health. 2005;4(1):53–62.

[22] Fleming PJ, Patel MR, Green M, Tariq M, Alhawli A, Syed N, Ali A, Bacon E, Godell S, Smith A, Harper D, Rescnicow K. Fear of Deportation and Associations with Mental Health Among Michigan Residents of Middle Eastern & North African Descent. J Immigr Minor Health. 2023;25(2):382–8. 10.1007/s10903-022-01394-w 36050543

[23] Johnson AL, Levesque C, Lewis NA, Asad AL. Deportation threat predicts Latino US citizens and noncitizens’ psychological distress, 2011 to 2018. Proc Natl Acad Sci U S A. 2024;121(9): e2306554121. 38377187 10.1073/pnas.2306554121 PMC10907276

[24] Menzi P, Rohner B. Handbuch Psychiatrische Versorgung im Freiheitsentzug. Schweizerisches Kompetenzzentrum für den Justizvollzug; 2021. Verfügbar unter: https://www.skjv.ch/de/handbuch/Psychiatrische-Versorgung

[25] Simpson AIF, Gerritsen C, Maheandiran M, Adamo V, Vogel T, Fulham L, Kitt T, Forrester A, Jones RM. A systematic review of reviews of correctional mental health services using the STAIR framework. Front Psychiatry. 2022;12:2515–2515. 10.3389/fpsyt.2021.747202 PMC8806032 35115956

[26] Enggist S, Møller L, Galea G, Udesen C. Prisons and Health [Internet]. World Health Organization, Herausgeber. 2014. 1 S. Verfügbar unter: https://apps.who.int/iris/bitstream/handle/10665/128603/PrisonandHealth.pdf;jsessionid=9D8EB2F358676D0942F6EA53A5307F94?sequence=1

[27] Harris PA, Taylor R, Minor BL, Elliott V, Fernandez M, O’Neal L, McLeod L, Delacqua G, Delacqua F, Kirby J, Duda SN. The REDCap consortium: Building A. international community of software platform partners. J Biomed Inf. 2019;95:103208–103208. 10.1016/j.jbi.2019.103208 PMC7254481 31078660

[28] von Elm E, Altman DG, Egger M, Pocock SJ, Gøtzsche PC, Vandenbroucke JP. The strengthening the reporting of observational studies in epidemiology (STROBE) statement: guidelines for reporting observational studies. Lancet 20 Oktober. 2007;370(9596):1453–7. 10.1016/S0140-6736(07)61602-X 18064739

[29] Derogatis LR. Brief symptom inventory. Third edit. Minneapolis: National Computer Services; 1993.

[30] Franke GH. Brief-Symptom-Checklist (BSCL). 1. Aufl. Göttingen: Hogrefe Verlag GmbH; 2017.

[31] Gonçalves LC, Dirkzwager AJE, Rossegger A, Gonçalves RA, Martins C, Endrass J. Mental and physical healthcare utilization among young prisoners: A longitudinal study. Int J Forensic Ment Health. 2017;16(2):139–48.

[32] Naumova K, Kitkanj Z. Approach/avoidance personality traits as predictors of psychopathology in convicted offenders. Psihologija. 2018;52(1):75–91.

[33] Dudeck M, Lathan M, Drenkhahn K, Jäger S, Spitzer C, Freyberger HJ, Franke GH. Eine Kurzversion des Brief Symptom Inventory (BSI-25-F) zum Einsatz bei Gefangenen im Langzeitstrafvollzug in Europa. Z Psychiatr Psychol Psychother. 2014;62(3):201–9.

[34] Wickham H, Averick M, Bryan J, Chang W, McGowan L, François R. u. A. Welcome to the tidyverse. J Open Source Softw. 2019;4(43):1686.

[35] Mazza GL, Enders CK, Ruehlman LS. Addressing item-level missing data: a comparison of proration and full information maximum likelihood estimation. Multivar Behav Res. 2015;50(5):504–504. 10.1080/00273171.2015.1068157 PMC4701045 26610249

[36] Wilson EB. Probable inference, the law of succession, and statistical inference. J Am Stat Assoc. 1927;22(158):209–12.

[37] Brown LD, Cai TT, DasGupta A. Interval estimation for a binomial proportion. Stat Sci. 2001;16(2):101–33.

[38] Stevenson M, Package. „epiR. Tools for the Analysis of Epidemiological Data. [Internet]. 2023 Sep. Report No.: version 2.0.65. Verfügbar unter: https://cran.r-project.org/web/packages/epiR/index.html

[39] Hosmer DW, Lemeshow S, Sturdivant RX. Applied Logistic Regression. 3rd Aufl. Bd. 398. Wiley; 2013. 528 S.

[40] Hemmert GAJ, Schons LM, Wieseke J, Schimmelpfennig H. Log-likelihood-based Pseudo-R2 in logistic regression: deriving Sample-sensitive benchmarks. Sociol Methods Res 1 August. 2018;47(3):507–31.

[41] Hosmer DW, Hosmer T, Le Cessie S, Lemeshow S. A comparison of goodness-of-fit tests for the logistic regression model. Stat Med. 1997;16(9):965–80. 9160492 10.1002/(sici)1097-0258(19970515)16:9<965::aid-sim509>3.0.co;2-o

[42] le Cessie S, van Houwelingen JC. A goodness-of-fit test for binary regression models, based on smoothing methods. Biometrics. 1991;47(4):1267–82.

[43] R Core Team. R: A language and environment for statistical computing. Vienna, Austria: R Foundation for Statistical Computing. 2023 Okt. Verfügbar unter: https://www.r-project.org/

[44] Posit Team. RStudio: Integrated Development for R. PBC, Boston MA. RStudio; 2023. Report No.: version 2023.6.1.524. Verfügbar unter: https://posit.co/

[45] Signorell A. mult. al. DescTools: Tools for Descriptive Statistics. 2023. Report No.: version 0.99.23.

[46] Firke S, Denney B, Haid C, Knight R, Zadra J. janitor: Simple Tools for Examining and Cleaning Dirty Data. 2023. Report No.: version 2.2.0. Verfügbar unter: https://cran.r-project.org/web/packages/janitor/index.html

[47] Lumley T. survey: analysis of complex survey samples [Internet]. 2023. Verfügbar unter: https://r-survey.r-forge.r-project.org/survey/

[48] Harrell FEJ. rms: Regression Modeling Strategies. 2023. Verfügbar unter: https://github.com/harrelfe/rms

[49] Allaire J, Xie Y, Dervieux C, McPherson J, Luraschi J, Ushey K. rmarkdown: Dynamic Documents for R [Internet]. 2023. Report No.: R package version 2.25. Verfügbar unter: https://github.com/rstudio/rmarkdown

[50] Bedaso A, Ayalew M, Mekonnen N, Duko B. Global estimates of the prevalence of depression among prisoners: A systematic review and Mmeta-analysis. Depress Res Treat. 2020;(1):3695209. 10.1155/2020/3695209 10.1155/2020/3695209 PMC7718061 33294222

[51] Brown GP, Hirdes JP, Fries BE. Measuring the prevalence of current, severe symptoms of mental health problems in a Canadian correctional population: implications for delivery of mental health services for inmates. Int J Offender Ther Comp Criminol. 2015;59(1):27–50. 24146355 10.1177/0306624X13507040

[52] Dudeck M, Drenkhahn K, Spitzer C, Barnow S, Kopp D, Kuwert P. u. A. Traumatization A.d mental distress in long-term prisoners in Europe. Punishm Soc. 2011;13(4):403–23.

[53] Rose A, Trounson JS, Louise S, Shepherd S, Ogloff JRP. Mental health, psychological distress, and coping in Australian cross-cultural prison populations. J Trauma Stress. 2020;33(5):794–803. 32339357 10.1002/jts.22515

[54] Butcher E, Packham C, Williams M, Miksza J, Kaul A, Khunti K. u. A. Screening male prisoners for depression A.d A.xiety with the PHQ-9 A.d GAD-7 A. NHS healthcheck: patterns of symptoms A.d caseness threshold. BMC Psychiatry 9 September. 2021;21(1):446. 10.1186/s12888-021-03453-2 PMC8428050 34496806

[55] Bessler C, Stiefel D, Barra S, Plattner B. Psychische Störungen und kriminelle üRckfälle bei männlichen jugendlichen Gefängnisinsassen. Z Für Kinder- Jugendpsychiatrie Psychother. 2018;47(1):1–14. 10.1024/1422-4917/a000612 30156463

[56] Fazel S, Doll H, Långström N. Mental disorders among adolescents in juvenile detention and correctional facilities: A systematic review and metaregression analysis of 25 surveys. J Am Acad Child Adolesc Psychiatry. 2008;47(9):1010–9. 18664994 10.1097/CHI.ObO13e31817eecf3

[57] Beaudry G, Yu R, Perry AE, Fazel S. Effectiveness of psychological interventions in prison to reduce recidivism: a systematic review and meta-analysis of randomised controlled trials. Lancet Psychiatry September. 2021;8(9):759–73. 10.1016/S2215-0366(21)00170-X PMC8376657 34419185

[58] Livanou M, Furtado V, Winsper C, Silvester A, Singh SP. Prevalence of mental disorders and symptoms among incarcerated youth: A Meta-Analysis of 30 studies. Int J Forensic Ment Health 2 Oktober. 2019;18(4):400–14.

[59] Baier A, Fritsch R, Ignatyev Y, Priebe S, Mundt AP. The course of major depression during imprisonment – a one year cohort study. J Affect Disord. 2016;189:207–13. 26451505 10.1016/j.jad.2015.09.003

[60] Boyd A, Van de Velde S, Vilagut G, de Graaf R, O׳Neill S, Florescu S. u. A. Gender differences in mental disorders A.d suicidality in europe: results from A.large cross-sectional population-based study. J Affect Disord. 2015;173:245–54. 25462424 10.1016/j.jad.2014.11.002

[61] Drapalski AL, Youman K, Stuewig J, Tangney J. Gender differences in jail inmates’ symptoms of mental illness, treatment history and treatment seeking. Crim Behav Ment Health. 2009;19(3):193–206. 19533597 10.1002/cbm.733

[62] Rossegger A, Wetli N, Urbaniok F, Elbert T, Cortoni F, Endrass J. Women convicted for violent offenses: adverse childhood experiences, low level of education and poor mental health. BMC Psychiatry. 2009;9(1):81. 20028499 10.1186/1471-244X-9-81 PMC2804674

[63] Seedat S, Scott KM, Angermeyer MC, Berglund P, Bromet EJ, Brugha TS. u. A. Cross-National associations between gender A.d mental disorders in the world health organization world mental health surveys. Arch Gen Psychiatry. 2009;66(7):785–95. 19581570 10.1001/archgenpsychiatry.2009.36 PMC2810067

[64] Marchi M, Corbellini I, Vaccari E, Pingani L, Ferrari S, Amaddeo F. Mental health of transgender people in prison: a systematic review and meta-analysis. Int Rev Psychiatry. 2023;36(7):714–28. 10.1080/09540261.2023.2287680 39630188

[65] Lysell H, Runeson B, Lichtenstein P, Långström N. Risk factors for filicide and homicide: 36-Year National matched cohort study. J Clin Psychiatry. 2013;74(2):18403. 10.4088/JCP.13m08372 24345878

[66] Rihmer Z, Gonda X, Rihmer A, Fountoulakis KN. Suicidal and violent behaviour in mood disorders: A major public health problem. A review for the clinician. Int J Psychiatry Clin Pract 1 Januar. 2010;14(2):88–94. 10.3109/13651501003624712 24922467

[67] Verdolini N, Pacchiarotti I, Köhler CA, Reinares M, Samalin L, Colom F. u. A. Violent criminal behavior in the context of bipolar disorder: systematic review A.d meta-analysis. J Affect Disord. 2018;239:161–70. 30014956 10.1016/j.jad.2018.06.050

[68] Keller AS, Rosenfeld B, Trinh-Shevrin C, Meserve C, Sachs E, Leviss JA. u. A. Mental health of detained A.ylum seekers. Lancet. 2003;362(9397):1721–3. 14643122 10.1016/S0140-6736(03)14846-5

[69] Robjant K, Hassan R, Katona C. Mental health implications of detaining asylum seekers: systematic review. Br J Psychiatry. 2009;194(4):306–12. 19336779 10.1192/bjp.bp.108.053223

[70] Dezsö D, Konrad N, Seewald K, Opitz-Welke A. Implementation of a suicide risk screening instrument in a remand prison service in Berlin. Front Psychiatry. 2018;9(665). 10.3389/fpsyt.2018.00665 PMC6297671 30618858

