# Supplementary material for: Prevalence and risk factors of mental health symptoms of individuals in different detention settings: a cross-sectional study
Source: BMC Psychiatry. 2025 Sep 2;25:847. doi: 10.1186/s12888-025-07255-8 (PMC12403325; doi:10.1186/s12888-025-07255-8)
Supplement: Supplementary file 1 — Supplementary Material 1. [file 12888_2025_7255_MOESM1_ESM.docx]

**Supplementary Material to the Publication**

**Prevalence and Risk Factors of Mental health symptoms of Individuals in Different Detention Settings: A Cross-Sectional Study**

Schnyder, N.,^1,2^ Endrass, J.,^1,2,3^ Albrecht, J. N.,^1,2,4^ Dreyer, J.,^1^ Graf, M.^3^ Habermeyer, E.,^5^ Rossegger, A.^1,2,3^

^1^ Department of Forensic Psychology, University of Konstanz, Germany;

^2^ Office of Corrections, Department of Justice and Home Affairs of the Canton of Zurich, Switzerland;

^3^ Department of Forensic Psychiatry, University of Basel, Switzerland;

^4^ Child Development Center, University Children’s Hospital Zurich, University of Zurich, Switzerland;

^5^ Department of Forensic Psychiatry, University Hospital of Psychiatry Zurich, Zurich, Switzerland.

**Short title:** Mental health symptoms of Incarcerated Individuals

**Correspondence to**

Nina Schnyder

Justizvollzug und Wiedereingliederung | Forschung & Entwicklung

Direktion der Justiz und des Inneren | Kanton Zurich

Hohlstrasse 552

8090 Zurich

Switzerland

e-mail: [nina.schnyder@ji.zh.ch](mailto:nina.schnyder@ji.zh.ch)

**Supplementary Material 1. Additional information on settings and procedures**

**Survey 1: setting**

This survey took place at the pretrial and police detention facility "Prison Zurich West" (henceforth, PZW). The PZW is a newly constructed facility, opened its door in April 2022, and accommodates up to 124 adults and adolescents of any gender. All individuals have been very recently detained, usually a few hours ago. Most individuals are being held in provisional arrest for up to 48 hours. Therefore, the turn-over rate at the PZW is large. During provisional arrest, prosecution and police investigate whether the grounds for detention can be refuted or confirmed. If they seem to be refuted, the individual is released; if confirmed, the prosecution can file a request to the compulsory measures court for further pretrial detention (Schweizerische Strafprozessordnung (StPO), 2022).

The conditions in provisional arrest at the PZW are strict. Usually, detainees are, for 23 hours each day, in their cell. They can leave their cells to walk in the yard one hour each day and if they have visitors, an appointment with their attorney, or the prosecution. They have access to television, radio, books, and, upon request, a telephone but only very limited contact to peers, family, and friends. This makes interactions with the correctional officers very important. Correctional officers supervise and care for the detained individuals multiple times a day and upon request. However, the daily routine in the PZW is tightly structured and there is only little time for each detainee. Upon request, detained individuals have access to primary health care as well as pastoral care of different religions.

**Survey 2: setting**

This survey took place at ten pretrial, correctional, and administrative detention centers in the Canton of Zurich. There are separate correctional facilities for longer sentences (1.5 years or longer) and shorter sentences (up to 1.5 years). All individuals have access to primary and specialised health care, such as dental care as well as mental health care. Furthermore, they have access to pastoral care of different religions. Correctional officers and other prison staff are constantly interacting with and caring for all detainees. Libraries, televisions, and restricted internet access are available in all facilities.

Individuals in pretrial detention are awaiting sentencing or trial. The time spent in pretrial detention is limited to three months. However, it is possible to extend this period – theoretically indefinitely. For up to eight hours a day, individuals are outside their cells. Contact to families and friends is limited, but visitations and phone calls are possible after approval by the state attorney. In one of the six pretrial detention facilities, most of the detainees are able to work, which gives them a daily routine. In all the other facilities, there is not enough work for most of the detainees. However, during the time outside their cells, detainees can interact with each other, do sports, or take courses.

Individuals in correctional detention have been convicted. During the day, individuals spent most of their time outside their cells and work. In the correctional facility for longer sentences it is possible to complete a vocational training. In all correctional facilities it is possible to learn a language or attend other courses. In their free time they can have contact with families and friends, interact with other detainees, or do sports.

Individuals in administrative detention are not convicted of a criminal offence but are awaiting deportation from Switzerland because they have violated the immigration law. During most of the day, individuals in administrative detention can freely move around the facility, interact with others, do sports, and work.

**Procedure both surveys**

We had been trained by the correctional staff in how to approach the detainees. We visited each cell with eligible individuals. Aims and scope of the study were explained verbally (German, English, or French) and in writing. It was also explained that participation is voluntary and that no negative or positive consequences can arise from non-participation. If consent was given verbally, we distributed the study documents consisting of study information, informed consent form, and paper-pencil-questionnaire (see *online Material 2* for the English version of the documents). The paper-pencil-questionnaire did not contain any person identifying information. The completion of the study documents was done in private with us being available to answer question. Detainees could withdraw their verbal consent anytime. Upon collection of the study documents, we examined whether participants had completed the informed consent form and questionnaires. If they had not and were not willing to do so, we destroyed these study documents.

**Additional procedure survey 1**

We informed that participants’ data would be made anonymous before their eyes upon collection of the study documents. As a reward for participation, we promised a small chocolate bar or candy. Completed informed consent forms and questionnaires were each packed in separate envelopes and sealed. Our sampling procedure made sure that an individual would not participate multiple times in the survey: From Tuesday to Thursday, we approached inmates who had been incarcerated the previous day; on Monday, we approached inmates who had been incarcerated the previous two days, and on Friday, we approached inmates who had been incarcerated the previous or the present day. This allowed us to approached eligible individuals who had been detained on the weekends.

**Additional procedure survey 2**

The procedure regarding where individuals were approached slightly varied between facilities. Individuals were either approached at their work place in the facility, in their cells, or, when the cell doors were open, on the corridors and yards. Data was pseudonymised at our office with a code key. Only selected individuals of our study group have access to the code key. Participants were informed about that procedure.

**eTable 1.** Individuals who answered sufficient number of items in each dimension and for the global severity index (GSI) (total N = 951)

| Dimensions and GSI | # Items per dimension | Individuals with sufficient data, n (%) | Individuals with insufficient data, n (%) |
| --- | --- | --- | --- |
| Anxiety | 6 | 943 (99.2%) | 8 (0.8%) |
| Depression | 6 | 924 (97.2%) | 27 (2.8%) |
| Hostility | 5 | 933 (98.1%) | 18 (1.9%) |
| Interpersonal sensitivity | 4 | 941 (98.9%) | 10 (1.1%) |
| Obsessive-compulsive | 6 | 939 (98.7%) | 12 (1.3%) |
| Paranoid ideation | 5 | 937 (98.5%) | 14 (1.5%) |
| Phobic anxiety | 5 | 939 (98.7%) | 12 (1.3%) |
| Psychoticism | 5 | 933 (98.1%) | 18 (1.9%) |
| Somatic complaints | 7 | 930 (97.8%) | 21 (2.2%) |
| *Additional* | 4 | 946 (99.5%) | 5 (0.5%) |
| Global Severity Index (GSI) | 53 | 951 (100%) | 0 (0%) |

*Note* The results regarding the BSCL dimensions should only be interpreted if no more than one item per dimension is missing; those regarding the GSI should only be interpreted if in total no more than 13 missing items are missing.

| **eTable 2.** Language of questionnaire (N=951) | | |
| --- | --- | --- |
|  |  | **% (n)** |
| Language of questionnaire | Albanian | 3.5% (33) |
|  | Arabic | 15.9% (151) |
|  | Croatian | 1.1% (10) |
|  | English | 9.4% (89) |
|  | French | 4.7% (45) |
|  | German | 49.0% (466) |
|  | Italian | 3.3% (31) |
|  | Macedonian | 0.1% (1) |
|  | Portuguese | 1.5% (14) |
|  | Romanian | 3.4% (32) |
|  | Serbian | 2.8% (27) |
|  | Spanish | 4.4% (42) |
|  | Turkish | 1.1% (10) |

**eTable 3.** Association between type of incarceration and case of mental health symptoms (N=951): estimates for the unadjusted model and for all variables of the fully adjusted model

|  |  | **OR** | **Lower**  **95%-UI** | **Upper**  **95%-UI** |
| --- | --- | --- | --- | --- |
| **Unadjusted model** |  |  |  |  |
| Type of detention | administrative detention | 3.645 | 1.588 | 8.614 |
|  | pretrial detention | 1.349 | 0.861 | 2.114 |
|  | provisional arrest | 3.628 | 2.541 | 5.239 |
|  | shorter incarceration | 1.550 | 0.887 | 2.687 |
| **Fully adjusted model** |  |  |  |  |
| Type of detention | administrative detention | 4.842 | 1.926 | 12.504 |
|  | pretrial detention | 1.509 | 0.924 | 2.474 |
|  | provisional arrest | 4.294 | 2.877 | 6.506 |
|  | shorter incarceration | 1.584 | 0.859 | 2.900 |
| Age categories in years | <20 | 1.542 | 0.755 | 3.203 |
|  | 30-39 | 1.010 | 0.708 | 1.439 |
|  | 40-49 | 0.732 | 0.476 | 1.123 |
|  | 50-59 | 0.620 | 0.345 | 1.092 |
|  | >= 60 | 2.125 | 1.006 | 4.557 |
| Gender | female | 1.679 | 0.935 | 3.079 |
|  | non binary | 3.943 | 1.088 | 18.895 |
| Previous treatment for mental health symptoms | yes | 3.806 | 2.790 | 5.228 |

Notes. OR: Odds Ratio, UI: uncertainty interval. Reference categories: longer incarceration, age 20-29, male, no previous treatment for mental health symptoms. McFadden Pseudo R2 (adjusted model)=0.135; le Cessie-van Houwelingen test p=0.950; Wald test: all covariates significantly contribute to model fit.

References

Schweizerische Strafprozessordnung (StPO), (2022). https://www.fedlex.admin.ch/eli/cc/2010/267/de
